# Supplementary material for: A construction method of reverberation suppression filter using an end-to-end network
Source: PLoS One. 2023 Oct 24;18(10):e0293365. doi: 10.1371/journal.pone.0293365 (PMC10597487; doi:10.1371/journal.pone.0293365)
Supplement: S1 File — (https://doi.org/10.6084/m9.figshare.24151551). (DOCX) [file pone.0293365.s001.docx]

S1 File. All data files are available from the figshare database.

(https://doi.org/10.6084/m9.figshare.24151551).
