# Supplementary material for: A construction method of reverberation suppression filter using an end-to-end network
Source: PLoS One. 2023 Oct 24;18(10):e0293365. doi: 10.1371/journal.pone.0293365 (PMC10597487; doi:10.1371/journal.pone.0293365)
Supplement: Attachment — Submitted filename: Response to Reviewers.docx [file pone.0293365.s002.docx]

**Original Manuscript ID:** PONE-D-23-16198

**Original Article Title:** “A construction method of reverberation suppression filter using an end-to-end network”

**To:** PLOS ONE Editor

**Re:** Response to reviewers

Dear Editor,

Thank you for allowing a resubmission of our manuscript, with an opportunity to address the reviewers’ comments.We will be happy to edit the text further, based on helpful comments from the reviewers.

We are uploading (a) our point-by-point response to the comments (below) (Response to Reviewers), (b) an updated manuscript with yellow highlighting indicating changes(Revised Article with Changes Highlighted), and (c) a clean updated manuscript without highlights (Manuscript).

Best regards,

<Zhen Wang> et al.

**Reviewer#1, Concern # 1:** The Reverberation Suppression Network (RS-U-Net) proposed in this article is used to suppress the reverberation of the underwater echo signal, effectively improving the signal-to-noise ratio of the echo signal. But there are several glaring problems in the current manuscript.

**Author response:** Thank you for your recognition of our previous research. I rewrote the experimental part by adding similar U-shaped network experiments (Wave-U-net, SEW-U-Net) to highlight the effectiveness of reverberation suppression.

**Reviewer#1, Concern # 2:** .The RS-U-Net structure proposed in the article is an improvement of the U-shaped network. The general structure of the network is similar to the end-to-end filter Wav-U-net and Demucs. It is recommended that the article focus on describing the advantages of RS-U-Net in suppressing reverberation. The article needs to highlight the innovations, and focus on a detailed description of the innovations, such as explaining the proposed RS-U-Net structure, the sonar echo signal processing method, etc

**Author response:** Thank you for your valuable suggestions. We have updated this paper, focusing on the advantages of RS-U-Net in suppressing reverberation and focusing on the innovation of the detailed description.

**Reviewer#1, Concern # 3:** There are too many pictures, it is recommended to combine the same type of pictures into one large picture, so that readers can understand and compare the pictures.

**Author response:** I am sorry for the confusion. We have updated the manuscript to combine the same type of figures into one large figure.

**Reviewer#1, Concern # 4:** The empirical formula of shallow sea noise spectrum level has different expressions at different levels, so there should be no universally recognized expression formula up to now, especially in the environment such as shallow sea acoustic noise, which has a certain range of recognition and rationality. It is suggested to explain the basis and source of the formula.

**Author response:** Thank you for your valuable suggestion. We have labeled the basis and source of the equation and explained the use of the equation in detail.

**Reviewer#1, Concern # 5:** Why use simulated data instead of real data? This kind of underwater acoustic experimental data should not be difficult to obtain, is there too few useful samples or the experimental conditions are not allowed?

**Author response:** Thank you for your valuable suggestion. Data collection for pool experiments still requires some money and the cooperation of a team, although it is less expensive than ocean experiments. The main reasons are:

1. It is not easy to label experimental data. The collected data will have strong reverberation and cannot be accurately labeled.

2. Versatility of the method. The ocean experiment will follow the pool experiment, and it is necessary to ensure that the method is common in the ocean experiment.

3. The device status needs to be satisfied. The device needs to be adjusted differently in different environments. In this case, adequate data cannot be obtained.

**Reviewer#1, Concern # 6:** RS-U-Net is little difference from the originalU-Net network, and the significant innovation part is not fully reflected on ‘RS’.

**Author response:** I'm sorry for the confusion. We have revised the whole article according to your request. The embodiment of "RS" is emphasized.

**Reviewer#2, Concern # 1:** Figures lack clarity.

**Author response:** Thank you for your valuable suggestions. We have updated the manuscript's figures to make them clear and more accessible for readers to read.

1) **Reviewer#2, Concern # 2:** Gramatical issues needs to be properly addressed.

**Author response:** Thank you for your valuable suggestion. We have updated the manuscript and solved the grammatical issues.

**Reviewer#2, Concern # 3:** Heading 1 is relate work. Please correct to related work

**Author response:** Thank you for your valuable suggestion. We have updated the manuscript to correct the heading "relate work" to "related work".

**Reviewer#2, Concern # 4:** Equation 3 and 4 needs more explaination. What is B in equation 4

**Author response:** Thank you for your valuable suggestion. We have updated the manuscript to give a more detailed explanation of Equation 3,4 and an explanation of "B" in the formula.

**Reviewer#2, Concern # 5:** Few figures are not properly explained.

**Author response:** Thank you for your valuable suggestion. We have updated the manuscript and the picture interpretation content to make it properly explained.

**Reviewer#2, Concern # 6:** Table 2 shows the experimental result that need to be further elaborated..

**Author response:** Thank you for your valuable suggestion. We updated the manuscript and further elaborated on the experimental results in Table 2

**Reviewer#2, Concern # 6**: A separate section experimental setup must be added

**Author response:** Thank you for your valuable suggestion. We updated the manuscript, added the experimental setting part, and introduced the experimental setting in detail.

**Thanks again for your advice and I hope to learn more from you.**
