# Supplementary material for: A construction method of reverberation suppression filter using an end-to-end network
Source: PLoS One. 2023 Oct 24;18(10):e0293365. doi: 10.1371/journal.pone.0293365 (PMC10597487; doi:10.1371/journal.pone.0293365)
Supplement: Attachment — Submitted filename: Response to Reviewers.docx [file pone.0293365.s003.docx]

**Original Manuscript ID:** PONE-D-23-16198R1

**Original Article Title:** “A construction method of reverberation suppression filter using an end-to-end network”

**To:** PLOS ONE Editor

**Re:** Response to reviewers

Dear Editor,

Thank you for allowing a resubmission of our manuscript, with an opportunity to address the reviewers’ comments.We will be happy to edit the text further, based on helpful comments from the reviewers.

We are uploading (a) our point-by-point response to the comments (below) (Response to Reviewers), (b) an updated manuscript with yellow highlighting indicating changes(Revised Article with Changes Highlighted), and (c) a clean updated manuscript without highlights (Manuscript).

Best regards,

<Zhen Wang> et al.

**Reviewer#1, Concern # 1:** In the described RS-U-Network construction section, we noticed the lack of elaboration on why 1D convolutions are used instead of 2D convolutions in network blocks. Considering that the use of different types of convolution operations in different fields will produce different effects, it is recommended to add a description in this section to explain why one-dimensional convolution is selected in the signal feature extraction process instead of two-dimensional convolution.This supplemental explanation can provide the reader with a deeper understanding of why 1D convolution is considered a more appropriate choice in a particular case.

**Author response:** Thank you for your recognition of our previous research.I have added a section explaining the advantages of choosing 1D convolution over 2D convolution during signal feature extraction and the advantages of 1D convolution.

**Reviewer#1, Concern # 2:** The RS-U-Net structure proposed in the article is improved on the basis of the U-shaped network, and its focus is mainly on the detailed description of the RS-U-Net structure. However, in the RS-U-Network Construction section, the way the article is presented may cause readers to be confused, because Network Blocks are introduced first, and then Network Structure is introduced. Therefore, it is recommended to reorganize the content in this part. First, a general introduction to the overall network structure is given, and then the specific content of each network module is discussed in depth.

**Author response:** Thank you for your valuable suggestions. I reorganized this part of the content. First, I gave a brief introduction of the whole network structure, and then I discussed the specific content of each main network module in depth.

**Reviewer#1, Concern # 3:** In the article, most of the references cited are from many years ago. It is recommended that the article cite the literature in recent years as much as possible to show that the method proposed in the article is closely related to the current research dynamics. New research results, new technologies and methods may emerge in the field of signal processing, which may be more powerful for supporting the discussion and method proposal of the article.

**Author response:** I am sorry for the confusion. I have updated the literature in the article and quoted the recent literature as much as possible.

**Reviewer #2:**The paper updated as requested. Necessary changes have been made. Presentation of the paper is improved.

**Author response:** Thank you for your suggestions. All your suggestions are very important and have important guiding significance for my thesis writing and scientific research work.

**Thanks again for your advice and I hope to learn more from you.**
